# Serotonin reuptake inhibitors improve muscle stem cell function and muscle regeneration in male mice: antidepressant effects beyond the brain

**DOI:** 10.1192/j.eurpsy.2025.1819

**Published:** 2025-08-26

**Authors:** M. Fefeu, M. Blatzer, A. Kneppers, D. Briand, P. Rocheteau, A. Haroche, D. Hardy, M. Juchet Martin, A. Danckaert, F. Coudoré, A. Tutakhail, C. Huchet, A. Lafoux, R. Mounier, O. Mir, R. Gaillard, F. Chrétien

**Affiliations:** 1 Child and Adolescent Psychiatry Department and the Child Brain Institute, Hôpital Robert Debré, AP- HP; 2 Institut Pasteur, Paris; 3 Institut NeuroMyoGène, Lyon; 4 Hôpital Sainte Anne, GHU Paris Psychiatrie Neurosciences, Paris; 5 Université Paris Saclay, Chatenay-Malabry; 6 Université Nantes, Nantes; 7 Gustave Roussy, Villejuif, France

## Abstract

**Introduction:**

Fluoxetine, the first selective serotonin reuptake inhibitor, is the world’s most prescribed antidepressant. Several mechanisms of action underpin the effect of this antidepressant, such as enhancing serotonin (5-HT) neurotransmission, increasing hippocampal neurogenesis, neuronal survival and cerebral angiogenesis. The effects of fluoxetine on stem cell behaviour and tissue regeneration beyond the central nervous system have been little studied to date.

**Objectives:**

We investigated whether fluoxetine (FLX) might have broader peripheral regenerative properties using a recognized regenerative medicine paradigm such as the animal model of ad integrum muscle regeneration.

**Methods:**

To investigate the impact of fluoxetine (FLX) on muscle at steady state, FLX was delivered *per os* at 18 mg/kg daily for six weeks to uninjured wild-type and specific transgenic mice. To investigate FLX´s regenerative capacity on skeletal muscles, we delivered FLX for six weeks and then performed notexin-induced injuries (phospholipase that induces a severe muscle necrosis) of the *tibialis anterior* muscle in wild-type and specific transgenic mice. Muscle force, muscle stem cells number, dividing muscle stem cells, differentiating muscle stem cells number, vessels number and muscle fiber parameters were specifically assessed.

**Results:**

After prolonged administration (6 weeks) of fluoxetine to male mice, we showed that prolonged FLX treatment increased the number of muscle stem cells and muscle angiogenesis in mice. FLX also improved skeletal muscle regeneration after single and multiple injuries induced by intramuscular notexin injection. The acceleration of muscle regeneration induced by FLX resulted from a triple action marked by an increase in the muscle stem cell pool, an increase in vessel density and a reduction in fibrotic lesions and inflammation. *In vitro*, we showed that the proliferative effects of FLX on immortalized myoblasts were dependent on 5-HT and 5-HT1B receptor activation. *In vivo*, mice lacking peripheral 5-HT treated with FLX did not show positive effects during muscle regeneration. Moreover, pharmacological, and genetic inactivation of the 5-HT1B receptor in muscle stem cells also abolished the FLX-induced improvement in muscle regeneration.

**Conclusions:**

We show that FLX promotes a harmonious muscle regeneration underpinned by a combined action on myogenesis, angiogenesis and inflammation. These results highlight the serotonergic identity of skeletal muscle and point to a promising therapeutic strategy for endogenous muscle diseases. Beyond muscle and brain, this work opens new perspectives of investigation both on the role of serotonin and the 5-HT1B receptor in other types of stem cells and on the therapeutic potential of antidepressants in regenerative medicine.

**Disclosure of Interest:**

None Declared

